# The Waxing, Waning, and Predictors of Humoral Responses to Vector-Based SARS-CoV-2 Vaccine in Hemodialysis Patients

**DOI:** 10.3390/vaccines10091537

**Published:** 2022-09-15

**Authors:** Chung-Ming Fu, Kai-Fan Tsai, Wei-Hung Kuo, Chien-Hsing Wu, Ching-I Yu, Huey-Ling You, Chien-Te Lee

**Affiliations:** 1 Division of Nephrology, Department of Internal Medicine, Kaohsiung Chang Gung Memorial Hospital and Chang Gung University College of Medicine, Kaohsiung 83301, Taiwan; 2 Department of Laboratory Medicine, Kaohsiung Chang Gung Memorial Hospital and Chang Gung University College of Medicine, Kaohsiung 83301, Taiwan

**Keywords:** anti-SARS-CoV-2 spike protein antibody, ChAdOx1 nCoV-19 vaccine, coronavirus disease 2019, end stage kidney disease, hemodialysis, severe acute respiratory syndrome coronavirus 2, vector-based SARS-CoV-2 vaccine

## Abstract

Hemodialysis (HD) patients are vulnerable to coronavirus disease 2019 (COVID-19) and have a high mortality rate. We evaluated the anti-SARS-CoV-2 spike protein antibody (ACOV2S) levels in 385 HD patients before and 4 and 8 weeks after the second dose of vector-based ChAdOx1 nCoV-19 vaccine. For study control, week 4 ACOV2S levels after the second vaccination dose were measured in 66 healthcare workers (HCWs). The seroconversion rate of HD patients was 98.96% 4 weeks after the second vaccination. Despite low antibody levels before the second dose (week 0), week 4 ACOV2S levels after the second vaccine dose in HD patients increased prominently and were compatible with those in HCWs (*p* = 0.814 for HCWs vs. HD patients). The ACOV2S levels in HD patients waned significantly 8 weeks after the second vaccination dose (*p* < 0.001 at week 8 vs. 4). Older age and immunosuppressant use were negative predictors, while higher C-reactive protein (CRP) levels were positive predictors of ACOV2S waxing after the second vaccine dose in HD patients. Higher CRP levels and platelet counts were independently associated with decreased ACOV2S waning. The ChAdOx1 nCoV-19 vaccine is effective and safe for primary vaccination in HD patients and a booster dose is necessary.

## 1. Introduction

The coronavirus disease 2019 (COVID-19), caused by the severe acute respiratory syndrome coronavirus 2 (SARS-CoV-2), has infected more than 520 million people and claimed more than 6 million lives globally following the outbreak in Wuhan, China [1]. Chronic kidney disease (CKD) is a major public health issue in dealing with the pandemic. The global estimated prevalence of CKD approaches 13%, and patients with advanced CKD who require renal replacement therapy are estimated at between 4.9 and 7.0 million worldwide [2]. As a population susceptible to this contagious illness, dialysis patients are fragile and vulnerable to COVID-19 due to accelerated immunosenescence, chronic inflammation, and multiple comorbidities [3]. Several multicenter studies have documented a markedly higher mortality rate of COVID-19 in dialysis population, ranging between 17–30% [3,4,5]. In particular, hemodialysis (HD) patients have an increased risk of viral exposure because most of them have to visit HD units regularly, where numerous patients and medical staff are crowded together for several hours during each dialysis session [6,7]. In addition to infection control measures, such as wearing facemasks, hand hygiene, and quarantine policies, vaccination is one of the most important strategies against the COVID-19 pandemic and warrants more attention in the HD population.

The mRNA vaccines can effectively improve the outcomes of COVID-19 in the general population and dialysis patients [8,9,10]. However, with proven efficacy and safety in the general population, vector-based SARS-CoV-2 vaccines, such as the ChAdOx1 nCoV-19 vaccine, are also widely utilized, especially for primary vaccination, and considered more affordable and convenient to distribute than mRNA vaccines [11]. However, information about the effectiveness and efficacy of vector-based SARS-CoV-2 vaccines in the HD population is still sparse, and the clinical predictors of immune responses to these vaccines are not yet specifically elucidated in HD patients. Taiwan has the highest incidence and prevalence of end-stage kidney disease (ESKD), and HD is the main renal replacement therapy for patients with ESKD in Taiwan [12,13,14]. With the growing burden of ESKD, the role of vector-based SARS-CoV-2 vaccines in the HD population is a crucial issue in Taiwan and worldwide. In this prospective investigation, we assessed humoral responses after two-dose vector-based ChAdOx1 nCoV-19 vaccines in HD patients and evaluated the predictors of antibody waxing and waning in this population.

## 2. Materials and Methods

### 2.1. Enrollment of Study Cohort

Patients living in geographically different areas in Taiwan were recruited from the HD center of the Kaohsiung Chang Gung Memorial Hospital between August 2021 and December 2021. The inclusion criteria were as follows: (1) adult patients (≥20 years of age) receiving maintenance HD in the hospital for at least one month, (2) patients who were naïve to SARS-CoV-2 infection and vaccination, and (3) patients willing to have blood samples drawn for anti-SARS-CoV-2 antibody measurements. Patients who received a heterologous SARS-CoV-2 vaccination or SARS-CoV-2 vaccines other than ChAdOx1 nCoV-19 vaccine, those who switched the modality of renal replacement therapy to peritoneal dialysis, those who received de novo kidney transplantation, patients who did not complete the blood sampling schedule, and those who were transferred to other HD centers during the study period were excluded from the analysis. In addition, we also enrolled healthcare workers (HCWs) from the hospital who would receive two-dose ChAdOx1 nCoV-19 vaccines to measure their humoral responses after complete vaccination. The enrolled HCWs were naïve to SARS-CoV-2 infection and vaccination before enrollment. The study protocol was approved by the Institutional Review Board and Ethics Committee of Chang Gung Medical Foundation, Taipei, Taiwan (IRB No. 202101321B0), and adhered to the principles of the Declaration of Helsinki. Informed consent was obtained from all enrolled patients and HCWs.

### 2.2. Vaccination Schedule and Measurement of Humoral Response after Vaccination

During the study period, all vaccinated HD patients and HCWs received two doses of ChAdOx1 nCoV-19 vaccines (i.e., AZD-1222, Vaxzevria, or previously Oxford/AstraZeneca COVID-19 vaccine) with standard dosages according to the predetermined schedule of the Taiwan Central Epidemic Command Center (CECC). The vaccination was evaluated and performed by hospital medical teams in the HD center for the enrolled patients and the outpatient department for the HCWs. An extended two-dose interval of vaccination (i.e., ≥10–12 weeks) was adopted to adhere to the policy of the Taiwan CECC [15,16]. For each vaccinated HD patient, three blood samples for anti-SARS-CoV-2 spike protein antibody (ACOV2S) levels were collected (i.e., week 0, week 4, and week 8 levels corresponding to the levels before the second dose of vaccination, and 4 weeks and 8 weeks after the second dose of vaccination, respectively), and one blood sample for the ACOV2S level at enrollment was collected for each HD patient not receiving SARS-CoV-2 vaccination. Furthermore, the blood samples of the HCWs were examined for ACOV2S levels 4 weeks after the second vaccination dose.

The ACOV2S levels, which served as surrogates for humoral responses after vaccination in this study, were measured by using the Elecsys^®^ Anti-SARS-CoV-2-S immunoassay on a Roche Cobas e801 system (Roche Diagnostics, Basel, Switzerland). This one-step double-antigen sandwich assay was developed to quantitatively assess total anti-SARS-CoV-2 spike protein receptor-binding domain antibodies in human serum and plasma specimens and has been regarded as a reasonable alternative to neutralizing antibody testing [17,18]. Blood samples from all participants were collected in 5 mL plastic collection tubes containing spray-coated silica and polymer gel (BD, Franklin Lakes, NJ, USA) and stored at 4 °C. The analytical procedures for the ACOV2S levels in the blood samples were performed according to the manufacturer’s instructions, as provided in the Supplementary Materials. The results of the analyses were interpreted as the ACOV2S levels (U/mL). The limit of blank and the limit of detection for the assay was 0.30 U/mL and 0.40 U/mL, respectively. The linearity was between 0.40 and 250.00 U/mL (extensible to 25,000.00 U/mL with 1:100 dilution). The cutoff value of ACOV2S seroconversion was defined as ≥0.80 U/mL, with a sensitivity of 98.80% and specificity of 99.98%, according to the manufacturer’s instructions [19]. Notably, the assigned U/mL for the assay was equivalent to the binding antibody unit (BAU)/mL defined by the World Health Organization (WHO) International Standard for Anti-SARS-CoV-2 Immunoglobulin (NIBSC code 20/136) [17].

### 2.3. Collection of Patient Characteristics and Adverse Events after Vaccination

At enrollment, the demographic data of eligible patients were extracted from the electronic medical record system and HD records of the hospital, including age, sex, body mass index (BMI), dry body weight for HD treatment, HD vintage, diabetes, hypertension, chronic hepatitis B and C infections, systemic lupus erythematosus, transplant history, and use of immunosuppressants. The collected data were reviewed by two consultant nephrologists at the hospital. BMI and dry body weight were recorded one week before the first vaccination dose. Diabetes was defined as the persistent use of at least one glucose-lowering agent for more than one month or having at least two consecutive tests of glycated hemoglobin levels ≥ 6.5%. Hypertension was defined as the persistent use of at least one antihypertensive agent for more than one month or at least two blood pressure measurements above 140/90 mmHg. Chronic hepatitis B or C infection was recorded if a patient had two positive consecutive tests (interval > 6 months) for the hepatitis B surface antigen or hepatitis C antibody without confirmed successful viral eradication. The use of immunosuppressants within one month before the first dose of vaccination (such as steroids equivalent to prednisolone ≥ 5 mg/day, calcineurin inhibitors, mycophenolate mofetil, etc.) was also recorded. Other demographic profiles were collected from the medical records. Furthermore, clinical profiles within one month before the first dose of vaccination were collected for the eligible patients, including hematological profiles, indicators of dialysis adequacy, and blood biochemical data, such as blood urea nitrogen, serum creatinine, albumin, liver enzyme, total bilirubin, lipid and iron profiles, electrolytes, intact parathyroid hormone (intact-PTH), and C-reactive protein (CRP). For blood laboratory profiles, pre-dialysis data were selected midweek (Wednesday or Thursday) for patients undergoing HD three times a week or at the last dialysis session of the week (Friday or Saturday) for patients undergoing HD twice a week. Moreover, after each vaccination dose, adverse events in HD patients were investigated by the medical team of the HD center and categorized as localized or systemic events accordingly.

### 2.4. Statistical Analysis

Categorical variables are presented as numbers (*n*) with percentages. Continuous variables are presented as medians with interquartile ranges (IQRs) because of the non-normal distribution of most data assessed using the Kolmogorov–Smirnov method. For vaccinated patients, ACOV2S levels at weeks 0, 4, and 8 were compared using the Wilcoxon signed-rank test. The comparison of week 4 ACOV2S levels between vaccinated HD patients and HCWs was performed using the Mann–Whitney U test. The incidence of localized and overall systemic adverse events after the first and second doses of vaccination was compared using the McNemar’s test. To investigate the associations between humoral responses and adverse events after the second dose of vaccination, the week 4 ACOV2S levels of vaccinated HD patients were stratified by adverse events and compared using the Mann–Whitney U test. To identify the independent predictors of humoral responses and antibody waning after the second dose of vaccination, the effects of patient characteristics on the serial change in ACOV2S levels were examined by univariate and multivariate linear generalized estimating equation (GEE) analyses [20]. The logarithms of ACOV2S levels were utilized for the GEE analyses because of the right-skewed distribution of the antibody levels, and the multivariate analyses were adjusted for age, sex, BMI, diabetes, HD vintage, and all covariates with a *p*-value of <0.10 in univariate analyses using the enter method [21]. Statistical significance was set at a *p*-value of <0.05. All analyses were performed using the Statistical Product and Service Solutions (SPSS) software (version 22.0; IBM, Armonk, NY, USA).

## 3. Results

### 3.1. Characteristics of Enrolled Patients

**Figure 1:**
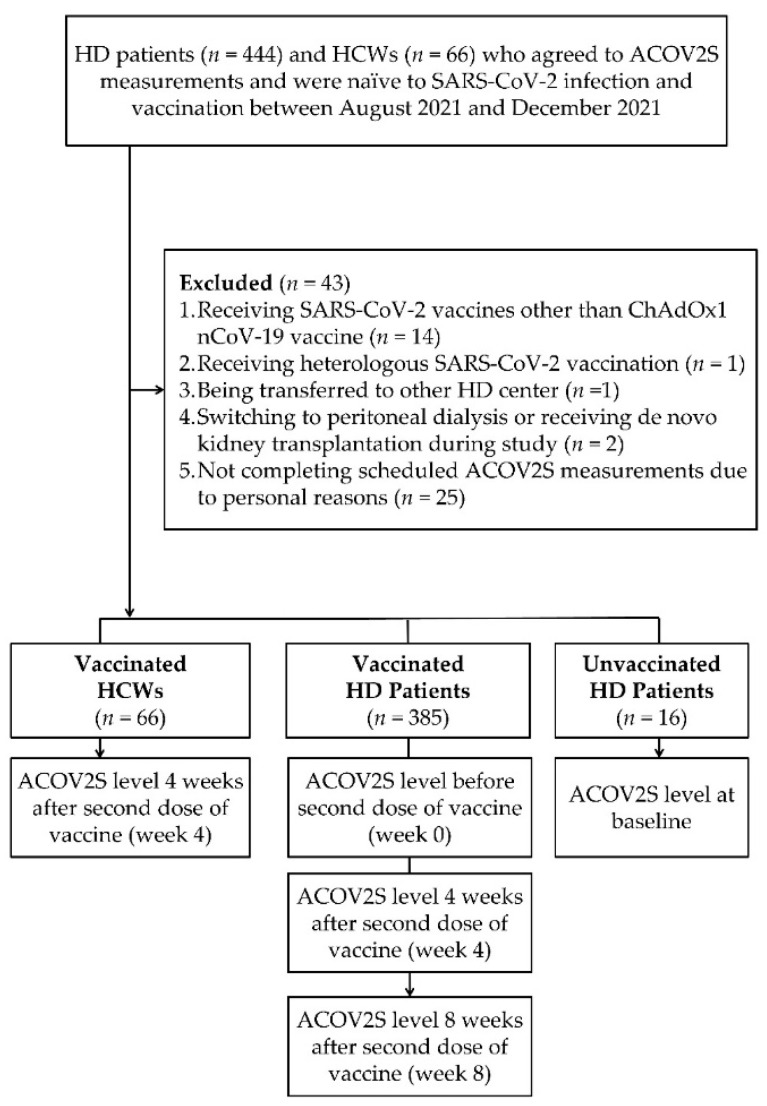
Enrollment of Study Cohort. ACOV2S, anti-SARS-CoV-2 spike protein antibody; HCW, healthcare worker; HD, hemodialysis; n, number; SARS-CoV-2, severe acute respiratory syndrome coronavirus 2.

We enrolled 444 adult patients undergoing maintenance HD twice or thrice per week. During the study period, 43 HD patients were excluded according to the prespecified exclusion criteria; 385 completely vaccinated and 16 unvaccinated HD patients were eligible for analysis. Three measurements of ACOV2S levels (i.e., week 0, week 4, and week 8) were collected for completely vaccinated patients, and the baseline ACOV2S levels were examined for unvaccinated patients. In addition, week 4 ACOV2S levels after the second vaccination dose were measured in 66 HCWs (Figure 1). During the study period, neither enrolled patients nor HCWs were confirmed to have SARS-CoV-2 infections. The characteristics of the vaccinated HD patients are presented in Table 1. The median age was 64 years (IQR, 55–70) and women accounted for 48.05% of the vaccinated patients, with a median HD vintage of 5.62 years (IQR, 2.56–14.31). The median BMI and dry body weight of the vaccinated patients were 22.10 kg/m^2^ (IQR, 19.50–24.70) and 58.00 kg (IQR, 51.00–66.60), respectively. Data showed that 52.21%, 35.58%, 14.29%, 8.05%, and 3.38% of vaccinated patients had hypertension, diabetes, chronic hepatitis B infection, chronic hepatitis C infection, and systemic lupus erythematosus, respectively. Furthermore, 15 (3.90%) vaccinated patients had a history of transplantation (kidney transplant, *n* = 13; liver transplant, *n* = 2), and 40 (10.39%) vaccinated patients of the cohort received immunosuppressants. The median age of the enrolled HCWs was 47 years (IQR, 40–51), and 90.91% of them were females.

### 3.2. Humoral Responses of HD Patients and HCWs after Vaccination

**Figure 2:**
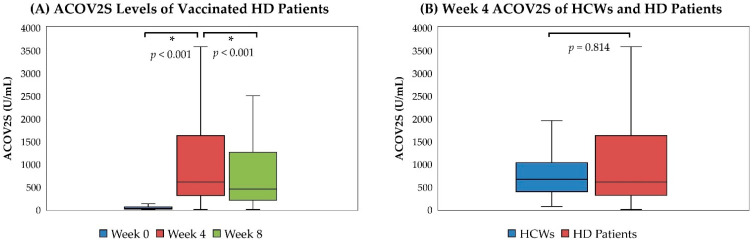
Humoral Responses of HD patients and HCWs after Second Dose of ChAdOx1 nCoV-19 Vaccine. (**A**) ACOV2S levels before and after second dose of vaccination in HD patients (*n* = 385); (**B**) comparison of week 4 ACOV2S levels between HCWs (*n* = 66) and HD patients (*n* = 385). *: *p* < 0.05.

The humoral responses of the 385 HD patients receiving complete two-dose vaccination are shown in Table 2 and Figure 2A. The interval between the two doses of ChAdOx1 nCoV-19 vaccines ranged from 89 to 118 days (median (IQR), 98 (98–100)) in vaccinated patients. The week 0 ACOV2S levels, which indicated humoral responses after the first dose of vaccination, demonstrated a seroconversion rate (≥0.80 U/mL) of 92.47% and a median titer of 23.10 U/mL (IQR, 7.30–56.60) in vaccinated patients. The week 4 ACOV2S levels after the second dose of vaccination revealed a remarkable increase of antibody titers compared with week 0 titers (median (IQR), 602.00 (307.50–1623.00) U/mL, *p* < 0.001 for week 4 vs. week 0) and a seroconversion rate of 98.96%. In contrast, week 8 ACOV2S levels after the second dose of vaccination waned significantly compared with week 4 levels (median (IQR), 449.00 (203.00–1258.00) U/mL, *p* < 0.001 for week 8 vs. week 4), but the seroconversion rate was maintained at 98.96%. The characteristics of patients without seroconversion after the first or second dose of vaccination, who accounted for a very small portion of the cohort, are listed in Supplementary Materials Table S1. On the other hand, the ACOV2S levels were all <0.40 U/mL in the 16 unvaccinated HD patients. In the 66 HCWs receiving complete vaccination, the two-dose interval of ChAdOx1 nCoV-19 vaccines ranged from 70 to 100 days (median (IQR), 74 (74–75)), and their week 4 ACOV2S levels after the second dose of vaccination were similar to those of HD patients (median (IQR), 662.50 (391.25–1029.25), *p* = 0.814 for HCWs vs. HD patients), along with a 100% seroconversion rate (Figure 2B).

### 3.3. The Adverse Events after Vaccination and Their Associations with Humoral Responses

**Figure 3:**
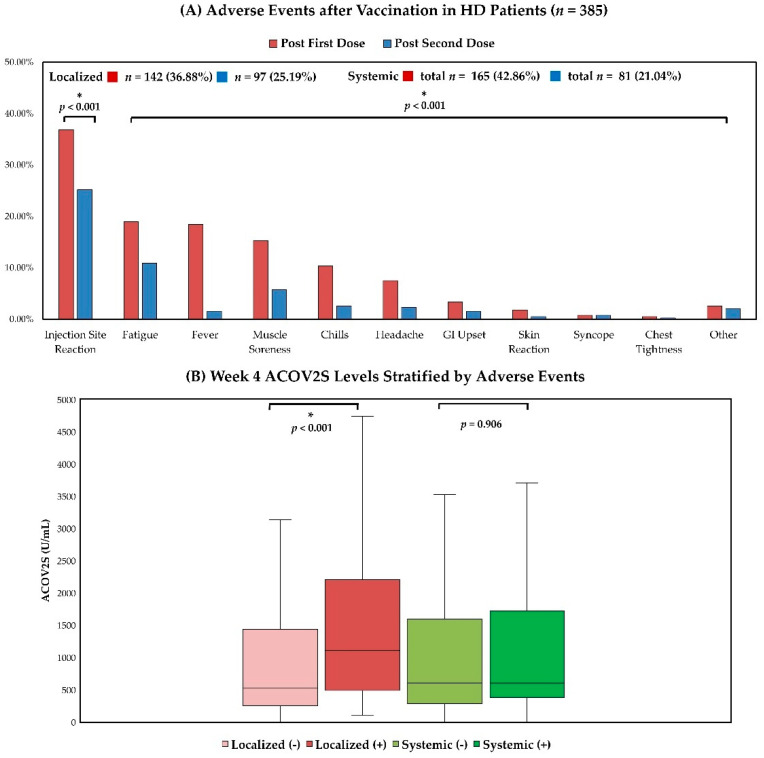
The Adverse Events after Vaccination and their Associations with Humoral Responses in HD Patients (*n* = 385) (**A**) Adverse events after vaccination in HD patients; (**B**) Week 4 ACOV2S levels stratified by adverse events after second dose of vaccination in HD patients. The adverse events after first and second doses of vaccination were as follows: injection site reaction, such as pain, redness, or swelling, *n* = 142 (36.88%) and 97 (25.19%); fatigue/dizziness, *n* = 73 (18.97%) and 42 (10.91%); fever, *n* = 71 (18.44%) and 6 (1.56%); muscle soreness/arthralgia, *n* = 59 (15.32%) and 22 (5.71%); chills, *n* = 40 (10.39%) and 10 (2.60%); headache, *n* = 29 (7.53%) and 9 (2.34%); GI upset, *n* = 13 (3.38%) and 6 (1.56%); skin reaction, such as rash, petechiae, or ecchymosis, *n* = 7 (1.82%) and 2 (0.52%); syncope/hypotension, *n* = 3 (0.78%) and 3 (0.78%); chest tightness, *n* = 2 (0.52%) and 1 (0.26%); and other events, such as rhinorrhea/pruritus/blurred vision/elevated blood pressure/insomnia, *n* = 12 (2.60%) and 8 (2.08%). GI, gastrointestinal. *: *p* < 0.05.

Adverse events after vaccination in the 385 patients undergoing HD are shown in Figure 3A. Localized and systemic adverse events after the first dose of vaccination were described by 36.88% and 42.86% of the vaccinated HD patients. The three most common systemic adverse events after the first vaccine dose were fatigue/dizziness (18.97%), fever (18.44%), and muscle soreness/arthralgia (15.32%). After the second dose of vaccination, 25.19% and 21.04% of vaccinated patients reported localized and systemic adverse events. The three most frequent systemic adverse events after the second vaccine dose were fatigue/dizziness (10.91%), muscle soreness/arthralgia (5.71%), and chills (2.60%). In addition, four hospitalization-requiring events occurred after the first dose of vaccination (syncope/hypotension, *n* = 2; chest tightness, *n* = 2), and three hospitalization-requiring events were recorded after the second dose of vaccination (syncope/hypotension, *n* = 2; chest tightness, *n* = 1) during the study period. All hospitalization-requiring events had full recovery after conservative management, and neither mortality nor other rare lethal events, such as vaccine-induced immune thrombocytopenia and thrombosis (VITT), were observed during the study period. Compared with the first vaccine dose, the enrolled patients reported fewer events after the second dose of vaccination in almost all categories, and the incidence rates of localized and overall systemic adverse events were significantly lower after the second vaccine dose (*p* < 0.001 for post-first dose vs. second dose for localized events and *p* < 0.001 for post-first dose vs. second dose for overall systemic events). The relationship between adverse events and humoral responses after the second vaccination dose is shown in Figure 3B. The HD patients with localized adverse events after second dose of vaccination had significantly higher week 4 ACOV2S levels compared with those without localized adverse events (median (IQR), with and without localized adverse events, 1105.00 (490.50–2204.50) and 523.00 (251.00–1437.00) U/mL, *p* < 0.001). On the other hand, the week 4 ACOV2S levels were similar between patients with and without systemic adverse events after second dose of vaccination (median (IQR), with and without systemic adverse events, 598.00 (378.50–1719.00) and 602.00 (285.00–1593.00) U/mL, *p* = 0.906).

### 3.4. Independent Predictors of Humoral Responses and Antibody Waning after Vaccination

In the 385 vaccinated HD patients, GEE analyses demonstrated that both week 4 and 8 ACOV2S levels increased significantly from week 0 levels, with elevations in ACOV2S of 1.5539 log U/mL (95% confidence interval (CI), 1.4791–1.6286, *p* < 0.001) and 1.3982 log U/mL (95% CI, 1.3248–1.4716, *p* < 0.001), respectively. Additionally, with the univariate and multivariate GEE analyses, age and immunosuppressant use were identified as independent predictors inversely correlated with the elevations of ACOV2S, with declines in ACOV2S of 0.0134 log U/mL per year of age (95% CI, −0.0071–−0.0197, *p* < 0.001) and 0.2546 log U/mL (95% CI, −0.0451–−0.4640, *p* = 0.017), respectively. Moreover, CRP level was an independent predictor positively associated with elevated ACOV2S, with an increase in ACOV2S of 0.0082 log U/mL per mg/L of CRP (95% CI, 0.0014–0.0149, *p* = 0.018) (Table 3). On the other hand, in the GEE analyses of serial ACOV2S levels, significant waning of antibodies was revealed between weeks 4 and 8 after the second dose of vaccination, with a decline in ACOV2S of 0.1558 log U/mL (95% CI, −0.1401–−0.1715, *p* < 0.001). Furthermore, univariate and multivariate GEE analyses recognized that higher CRP levels and platelet counts were independently associated with reduced antibody waning and with elevations in ACOV2S of 0.0137 log U/mL per mg/L of CRP (95% CI, 0.0059–0.0215, *p* = 0.001) and 0.0014 log U/mL per 10^9^/L of platelet count (95% CI, 0.0002–0.0025, *p* = 0.017), respectively (Table 4). 

## 4. Discussion

In this study, we obtained blood ACOV2S levels from 385 HD patients and demonstrated that these patients could reach a high seroconversion rate of 92.47% after the first dose of the ChAdOx1 nCoV-19 vaccine. Furthermore, the seroconversion rates remained as high as 98.96% at weeks 4 and 8 after the second vaccine dose, which were not inferior to those of dialysis patients receiving mRNA vaccines in previous studies [22,23,24,25,26]. While referring to the antibody levels, despite the low levels before the second dose of vaccination, the ACOV2S levels of HD patients increased markedly at week 4 after the second dose of ChAdOx1 nCoV-19 vaccine and were compatible with those of HCWs. Although HD patients receiving mRNA vaccines, such as the BNT162b2 or mRNA-1273 vaccine, were not enrolled in our investigation, the ACOV2S levels of these populations were assessed using the Elecsys^®^ Anti-SARS-CoV-2-S immunoassay in the literature. In a prospective cohort study, Simon et al. examined the humoral responses after the second dose of the BNT162b2 vaccine using the assay in 81 HD patients and the median ACOV2S level 21 days after the second dose of BNT162b2 vaccine was 171 U/mL [27]. Paal et al. retrospectively assessed 179 COVID-19-naïve HD patients 3–6 weeks after the second dose of mRNA vaccine (mainly BNT162b2 vaccine), and the median level of ACOV2S was 252.5 U/mL by the same assay [28]. In another cohort study utilizing the same assay, the median levels of ACOV2S were 297 U/mL in COVID-19-naïve HD patients receiving the BNT162b2 vaccine and 1032 U/mL in those receiving the mRNA-1273 vaccine; the median interval from the second dose of vaccination to ACOV2S tests was 48 days [29]. Hence, although previous studies have concluded that adenovirus vector-based SARS-CoV-2 vaccines induce lower immune responses than mRNA vaccines (especially the mRNA-1273 vaccine) [3,30,31,32], our study indicates that the humoral responses induced by the two-dose ChAdOx1 nCoV-19 vaccines might be non-inferior to those induced by the BNT162b2 vaccines in HD patients. Considering the easier deployment and more affordable cost, the vector-based ChAdOx1 nCoV-19 vaccine is an essential tool to ensure global SARS-CoV-2 vaccination coverage, and our findings support its role in primary vaccination in HD patients, although further head-to-head studies comparing vector-based vaccines and mRNA vaccines in this population are required.

Our research demonstrated that the ChAdOx1 nCoV-19 vaccine was well-tolerated in most HD patients. Of the 385 HD patients, four patients after the first dose of vaccination and three patients after the second dose of vaccination experienced hospitalization-requiring adverse events, but neither mortality nor VITT events were noted. In contrast to patients receiving mRNA vaccines whose adverse events are usually more prominent after the second vaccine dose [8,9,33,34,35], our study revealed that the adverse events were less frequent after the second dose of ChAdOx1 nCoV-19 vaccine in HD patients. Additionally, in our analysis, the occurrence of localized adverse events was positively associated with humoral responses four weeks after the second dose of vaccination in the HD population. Despite being reported in previous research on other vaccines, such as human papillomavirus and hepatitis E virus vaccines, the correlation between humoral responses and adverse events has not been studied in SARS-CoV-2 vaccines and therefore warrants further investigation [36].

There is a paucity of literature analyzing the waxing and waning of humoral responses in dialysis patients receiving the ChAdOx1 nCoV-19 vaccine. Despite the low levels after the first dose of the ChAdOx1 nCoV-19 vaccine, the ACOV2S levels increased significantly at week 4 after the second vaccine dose in most HD patients, and then waned at week 8 in our study. This dynamic change in humoral response is compatible with previous studies on dialysis patients immunized with other types of vaccines [32]. Using multivariate analyses, we found that older age and immunosuppressant use were independent predictors associated with poorly increased ACOV2S levels. Furthermore, CRP levels were positively correlated with ACOV2S elevation after the second dose of vaccination and inversely associated with antibody waning in our analysis. Published data support the hypothesis that older age and use of immunosuppressants have a remarkably negative impact on the development of humoral responses to vaccination in dialysis patients, which was also observed in our study [23,25,37,38,39]. Although previous studies indicated that dialysis patients with higher baseline CRP levels might have fewer humoral responses to vaccination [37,40,41], our analysis recognized that the elevation of ACOV2S levels after the second dose of ChAdOx1 nCoV-19 vaccine was more prominent in HD patients with higher baseline CRP levels, despite the low antibody levels after the first vaccine dose. Similarly, in a study of maintenance HD patients receiving the third dose of the BNT162b2 vaccine [42], Espi et al. observed that HD patients with lower humoral responses to primary two-dose vaccination appeared to derive more benefits from the third dose. They hypothesized that the higher humoral response to subsequent vaccination in these initially lower responders might be related to the presence of spike-specific T follicular helper cells. Although cellular immunity and T follicular helper cells were not assessed in our investigation, previous studies have identified a positive correlation between CRP and T follicular helper cell levels in patients with inflammatory diseases [43,44,45]. Further research is warranted to elucidate the relationship between CRP levels and humoral responses to SARS-CoV-2 vaccination in the HD population.

Additionally, a higher baseline platelet count was identified as an independent predictor of diminished antibody waning after the second dose of the ChAdOx1 nCoV-19 vaccine in our study. Although platelet is now recognized as an essential component of innate and adaptive immunity [46,47] and could be activated by SARS-CoV-2 spike proteins after vaccination and infection [48,49,50], its link with humoral responses to SARS-CoV-2 vaccination remains unevaluated. Decreased antibody waning in dialysis patients with higher baseline platelet counts may be multifactorial. Approximately 16–55% of patients with ESKD have reduced platelet counts [51], and the possible etiologies include medications, uremic toxins, blood loss during dialysis, use of heparin, nutritional disorders, and autoimmune diseases [52,53]. Moreover, hypocellular bone marrow is common in chronic dialysis patients [54]. Since various causes of thrombocytopenia might also impact humoral responses in vaccinated HD patients, further studies are required to clarify the role of platelets in humoral responses to SARS-CoV-2 vaccination in this population.

Our study had some limitations. Due to the single-center nature of the study, the sample size and racial diversity of subjects were inherently limited. All vaccinated patients received the same vector-based ChAdOx1 nCoV-19 vaccine as in the study design; hence, we could not directly compare the immunogenicity induced by different types of vaccines in the study population. The HCWs in our study were generally younger and predominantly female, and their detailed characteristics were unavailable. Moreover, baseline ACOV2S levels before the first dose of vaccination were not assessed in this study; hence, we could not exclude the possibility that stronger humoral responses in some individuals might be related to prior COVID-19 infection. However, during our study period, Taiwan had a very low COVID-19 prevalence due to meticulous quarantine measures [55,56,57], and the low prevalence could also be verified by the unmeasurable ACOV2S levels of all unvaccinated HD patients in our study. Finally, neither the biomarkers of cellular immunity nor humoral neutralizing ability were measured in our study. Although the correlations between ACOV2S levels, viral neutralization ability, and clinical protective efficacy after vaccination have been supported in the literature [58,59,60], the real-world efficacy of higher antibody titers in preventing severe SARS-CoV-2 infection in dialysis patients requires further research. Despite these limitations, our study highlights the role of the ChAdOx1 nCoV-19 vaccine as the primary SARS-CoV-2 vaccination in HD patients and demonstrates the clinical predictors of antibody waxing and waning after vaccination, which will hence be an informative reference for clinicians and investigators to optimize vaccination strategies against COVID-19 in this vulnerable population. Furthermore, the waning of antibodies at week 8 after the second dose of vaccination in our investigation supports the necessity of further vaccine boosters in the HD population.

## 5. Conclusions

In conclusion, our study indicates that the ChAdOx1 nCoV-19 vaccine is well tolerated in HD patients and could reach a high seroconversion rate after the first dose of vaccination as well as a humoral response compatible with that of the general population after the second vaccine dose. Moreover, older age and immunosuppressant use could negatively impact humoral responses after the second dose of vaccination, and CRP levels might be positively correlated with humoral responses after the second vaccine dose in the HD population. Considering the obvious waning of antibodies at week 8 after the second dose of vaccination, which might be inversely correlated with CRP levels and platelet count in our analysis, further vaccine boosters are required to prevent severe COVID-19 infection in the HD population.

## Figures and Tables

**Table 1:** Characteristics of HD Patients (*n* = 385) Receiving ChAdOx1 nCoV-19 Vaccine.

**Demographic Profiles**	
Age (year), median (IQR)	64 (55–70)
Female, *n* (%)	185 (48.05)
BMI (kg/m^2^), median (IQR)	22.10 (19.50–24.70)
Dry Body Weight (kg), median (IQR)	58.00 (51.00–66.60)
HD Vintage (year), median (IQR)	5.62 (2.56–14.31)
Urea Reduction Ratio, median (IQR)	0.74 (0.69–0.78)
Kt/V, median (IQR)	1.63 (1.39–1.86)
Diabetes, *n* (%)	137 (35.58)
Hypertension, *n* (%)	201 (52.21)
Chronic Hepatitis B, *n* (%)	55 (14.29)
Chronic Hepatitis C, *n* (%)	31 (8.05)
SLE, *n* (%)	13 (3.38)
Transplantation History ^#^, *n* (%)	15 (3.90)
Immunosuppressant, *n* (%)	40 (10.39)
**Clinical Profiles**	
Hemoglobin (g/L), median (IQR)	108.00 (101.00–114.00)
Leukocyte (10^9^/L), median (IQR)	6.10 (5.10–7.30)
Platelet (10^9^/L), median (IQR)	180.00 (147.00–222.50)
BUN (mmol/L), median (IQR)	22.85 (19.28–27.13)
SCr (μmol/L), median (IQR)	935.27 (811.07–1086.88)
Alb (g/L), median (IQR)	40.40 (38.40–42.05)
ALT (μkat/L), median (IQR)	0.20 (0.15–0.28)
Total Bilirubin (μmol/L), median (IQR)	5.13 (3.42–6.84)
Total Cholesterol (mmol/L), median (IQR)	4.07 (3.52–4.69)
Triglyceride (mmol/L), median (IQR)	1.30 (0.88–2.07)
Ferritin (μg/L), median (IQR)	308.10 (179.05–462.25)
Transferrin Saturation (%), median (IQR)	28.37 (22.25–35.90)
Potassium (mmol/L), median (IQR)	4.80 (4.30–5.20)
Total Calcium (mmol/L), median (IQR)	2.38 (2.25–2.53)
Phosphorus (mmol/L), median (IQR)	1.62 (1.36–1.89)
Intact-PTH (ng/L), median (IQR)	254.90 (73.35–550.00)
CRP (mg/L), median (IQR)	3.01 (1.24–6.56)

Alb, albumin; ALT, alanine aminotransferase; BMI, body mass index; BUN, blood urea nitrogen; HD, hemodialysis; CRP, C-reactive protein; IQR, interquartile range; *n*, number; Intact-PTH, intact parathyroid hormone; SCr, serum creatinine; SLE, systemic lupus erythematosus. ^#^: kidney transplant (*n* = 13); liver transplant (*n* = 2).

**Table 2:** Humoral Responses of HD Patients (*n* = 385) after Second Dose of ChAdOx1 nCoV-19 Vaccine.

ACOV2S Levels before and after Second Dose of Vaccination		
	ACOV2S (U/mL), Median (IQR)	Seroconversion (≥0.8 U/mL), *n* (%)
Week 0	23.10 (7.30–56.60)	356 (92.47)
Week 4	602.00 (307.50–1623.00)	381 (98.96)
Week 8	449.00 (203.00–1258.00)	381 (98.96)

ACOV2S, anti-SARS-CoV-2 spike protein antibody; SARS-CoV-2, severe acute respiratory syndrome coronavirus 2.

**Table 3:** Factors Associated with Humoral Responses after Second Dose of Vaccination in HD Patients.

	Univariate		Multivariate		
	β (95% CI)	*p-*Value	β (95%CI)	SE	*p-*Value
Week 0 ACOV2S (Log U/mL)	Reference		Reference		
Week 4 ACOV2S (Log U/mL)	1.5539 (1.4791–1.6286)	<0.001 *	1.5539 (1.4791–1.6286)	0.0381	<0.001 **
Week 8 ACOV2S (Log U/mL)	1.3982 (1.3248–1.4716)	<0.001 *	1.3982 (1.3248–1.4716)	0.0374	<0.001 **
Age (year)	−0.0142 (−0.0089–−0.0195)	<0.001 *	−0.0134 (−0.0071–−0.0197)	0.0032	<0.001 **
Female	0.0506 (−0.0708–0.1720)	0.414	0.0893 (−0.0452–0.2238)	0.0686	0.193
BMI (kg/m^2^)	−0.0007 (−0.0139–0.0125)	0.917	−0.0045 (−0.0173–0.0083)	0.0065	0.491
Diabetes	−0.0808 (−0.2098–0.0482)	0.220	−0.0631 (−0.1966–0.0704)	0.0681	0.354
HD vintage (year)	0.0025 (−0.0058–0.0107)	0.557	0.0028 (−0.0057–0.0113)	0.0043	0.518
Urea Reduction Ratio	0.2302 (−0.7168–1.1771)	0.634			
Kt/V	0.1032 (−0.0607–0.2670)	0.217			
Dry Body Weight (kg)	0.0008 (−0.0032–0.0047)	0.703			
Hypertension	−0.1019 (−0.2229–0.0192)	0.099 ^#^	−0.0633 (−0.1758–0.0492)	0.0574	0.270
Chronic Hepatitis B	0.1590 (−0.0001–0.3182)	0.050 ^#^	0.0894 (−0.0695–0.2483)	0.0811	0.270
Chronic Hepatitis C	0.0679 (−0.1622–0.2980)	0.563			
SLE	−0.1829 (−0.5521–0.1862)	0.331			
Transplantation History	−0.1983 (−0.5873–0.1907)	0.318			
Immunosuppressant	−0.2034 (−0.4246–0.0179)	0.072 ^#^	−0.2546 (−0.0451–−0.4640)	0.1069	0.017 **
Hemoglobin (g/L)	−0.0039 (−0.0089–0.0011)	0.125			
Leukocyte (10^9^/L)	0.0298 (−0.0031–0.0628)	0.076 ^#^	0.0042 (−0.0325–0.0410)	0.0187	0.821
Platelet (10^9^/L)	0.0018 (0.0009–0.0028)	<0.001 *	0.0007 (−0.0004–0.0018)	0.0006	0.206
BUN (mmol/L)	0.0108 (0.0010–0.0206)	0.031 *	0.0083 (−0.0043–0.0209)	0.0064	0.195
SCr (μmol/L)	0.0005 (0.0002–0.0008)	0.001 *	0.0001 (−0.0003–0.0006)	0.0002	0.552
Alb (g/L)	0.0080 (−0.0130–0.0289)	0.455			
ALT (μkat/L)	−0.2010 (−0.4511–0.0491)	0.115			
Total Bilirubin (μmol/L)	−0.0092 (−0.0250–0.0065)	0.251			
Total Cholesterol (mmol/L)	0.0520 (−0.0080–0.1119)	0.089 ^#^	0.0025 (−0.0577–0.0627)	0.0307	0.935
Triglyceride (mmol/L)	0.0253 (−0.0107–0.0612)	0.168			
Ferritin (10 μg/L)	−0.0004 (−0.0012–0.0005)	0.391			
Transferrin Saturation (%)	−0.0012 (−0.0073–0.0049)	0.704			
Potassium (mmol/L)	−0.0186 (−0.1236–0.0864)	0.728			
Total Calcium (mmol/L)	−0.0363 (−0.3543–0.2816)	0.823			
Phosphorus (mmol/L)	0.1274 (−0.0095–0.2644)	0.068 ^#^	−0.0712 (−0.2489–0.1065)	0.0907	0.432
Intact-PTH (10 ng/L)	0.0010 (−0.0001–0.0019)	0.078 ^#^	0.0002 (−0.0008–0.0012)	0.0005	0.694
CRP (mg/L)	0.0081 (0.0014–0.0148)	0.018 *	0.0082 (0.0014–0.0149)	0.0035	0.018 **

CI, confidence interval; SE, standard error. *: *p* < 0.05 in univariate analysis; ^#^: *p* < 0.1 in univariate analysis; **: *p* < 0.05 in multivariate analysis.

**Table 4:** Factors Associated with Antibody Waning after Vaccination in HD Patients.

	Univariate		Multivariate		
	β (95% CI)	*p-*Value	β (95% CI)	SE	*p-*Value
Week 4 ACOV2S (Log U/mL)	Reference		Reference		
Week 8 ACOV2S (Log U/mL)	−0.1558 (−0.1401–−0.1715)	<0.001 *	−0.1558 (−0.1401–−0.1715)	0.0080	<0.001 **
Age (year)	−0.0122 (−0.0060–−0.0183)	<0.001 *	−0.0067 (−0.0140–0.0005)	0.0037	0.070
Female	−0.0421 (−0.1813–0.0970)	0.553	−0.0160 (−0.1730–0.1410)	0.0801	0.841
BMI (kg/m^2^)	0.0001 (−0.0147–0.0149)	0.992	−0.0082 (−0.0225–0.0062)	0.0073	0.264
Diabetes	−0.0569 (−0.2091–0.0953)	0.464	−0.0500 (−0.2079–0.1079)	0.0806	0.535
HD vintage (year)	−0.0015 (−0.0108–0.0079)	0.760	−0.0012 (−0.0110–0.0086)	0.0050	0.810
Urea Reduction Ratio	−0.0556 (-1.1037–0.9924)	0.917			
Kt/V	0.0324 (−0.1486–0.2133)	0.726			
Dry Body Weight (kg)	0.0019 (−0.0024–0.0061)	0.390			
Hypertension	−0.0600 (−0.1989–0.0790)	0.397			
Chronic Hepatitis B	0.1584 (−0.0020–0.3187)	0.053 ^#^	0.0986 (−0.0544–0.2515)	0.0780	0.206
Chronic Hepatitis C	0.1453 (−0.1046–0.3953)	0.254			
SLE	−0.1843 (−0.5396–0.1709)	0.309			
Transplantation History	−0.1812 (−0.6343–0.2718)	0.433			
Immunosuppressant	−0.1759 (−0.4132–0.0613)	0.146			
Hemoglobin (g/L)	−0.0048 (−0.0103–0.0007)	0.085 ^#^	−0.0057 (−0.0114–0.0000)	0.0029	0.051
Leukocyte (10^9^/L)	0.0257 (−0.0098–0.0613)	0.156			
Platelet (10^9^/L)	0.0021 (0.0010–0.0031)	<0.001 *	0.0014 (0.0002–0.0025)	0.0006	0.017 **
BUN (mmol/L)	0.0133 (0.0018–0.0249)	0.024 *	0.0047 (−0.0100–0.0194)	0.0075	0.530
SCr (μmol/L)	0.0005 (0.0002–0.0009)	0.005 *	0.0004 (−0.0002–0.0009)	0.0003	0.211
Alb (g/L)	−0.0069 (−0.0290–0.0152)	0.542			
ALT (μkat/L)	−0.0356 (−0.2974–0.2262)	0.790			
Total Bilirubin (μmol/L)	−0.0072 (−0.0243–0.0099)	0.408			
Total Cholesterol (mmol/L)	0.0149 (−0.0554–0.0852)	0.678			
Triglyceride (mmol/L)	0.0491 (0.0063–0.0919)	0.024 *	0.0266 (−0.0147–0.0680)	0.0211	0.207
Ferritin (10 μg/L)	−0.0002 (−0.0011–0.0006)	0.603			
Transferrin Saturation (%)	−0.0012 (−0.0074–0.0051)	0.713			
Potassium (mmol/L)	−0.0200 (−0.1397–0.0996)	0.743			
Total Calcium (mmol/L)	−0.0099 (−0.3637–0.3439)	0.956			
Phosphorus (mmol/L)	0.1967 (0.0465–0.3469)	0.010 *	−0.0085 (−0.2140–0.1971)	0.1049	0.936
Intact-PTH (10 ng/L)	0.0014 (0.0002–0.0025)	0.019 *	0.0007 (−0.0004–0.0019)	0.0006	0.229
CRP (mg/L)	0.0154 (0.0080–0.0228)	<0.001 *	0.0137 (0.0059–0.0215)	0.0040	0.001 **

*: *p* < 0.05 in univariate analysis; ^#^: *p* < 0.1 in univariate analysis; **: *p* < 0.05 in multivariate analysis.

## Data Availability

All data generated in this study are available from the corresponding author (ctlee33@cgmh.org.tw) upon reasonable request due to the research regulations of the hospital.

## Supplementary Materials

The following supporting information can be downloaded at: https://www.mdpi.com/article/10.3390/vaccines10091537/s1, Analytical Procedures of Anti-SARS-CoV-2 Spike Protein Antibody (ACOV2S) Levels; Table S1. Characteristics of Patients without Seroconversion after First or Second Dose of Vaccination.
