# Supplementary material for: The Waxing, Waning, and Predictors of Humoral Responses to Vector-Based SARS-CoV-2 Vaccine in Hemodialysis Patients
Source: Vaccines (Basel). 2022 Sep 15;10(9):1537. doi: 10.3390/vaccines10091537 (PMC9502593; doi:10.3390/vaccines10091537)
Supplement: Supplementary file 1 [file vaccines-10-01537-s001.zip › vaccines-1896202-supplementary.pdf]

Analytical Procedures of Anti-SARS-CoV-2 Spike Protein Antibody (ACOV2S) Levels

Blood samples from all participants were analyzed using Elecsys® Anti-SARS-CoV-2-S immunoassay on a Roche Cobas e801 system (Roche Diagnostics, Basel, Switzerland). This one-step double-antigen sandwich assay was developed to quantify total anti-severe acute respiratory syndrome coronavirus 2 (SARS-CoV-2) spike protein receptor-binding domain (RBD) antibodies in human serum and plasma specimens. It primarily captures immunoglobulin G and may also detect immunoglobulins M and A. In short, each blood sample was incubated with a mixture of biotinylated and ruthenylated SARS-CoV-2 RBD recombinant antigens. In the presence of corresponding antibodies, double-antigen sandwich immune complexes were formed. These complexes bound to the solid phase through the interaction of biotin and streptavidin after the addition of streptavidin-coated microparticles. Further, the reagent mixture was transferred to a measuring cell, where microparticles were magnetically captured onto the electrode surface. After removing the unbound materials, electrochemiluminescence was applied and measured with a photomultiplier. The signal yield, proportional to the total anti-SARS-CoV-2 spike protein RBD antibody level in the specimen, was interpreted as the ACOV2S level (U/mL). The assay was standardized against an internal Roche Diagnostics anti-RBD monoclonal antibody mixture with a 1 nM concentration corresponding to 20.00 U/mL. The limit of blank and limit of detection for the assay was 0.30 U/mL and 0.40 U/mL, respectively. The linearity was between 0.40 U/mL and 250.00 U/mL (extensible to 25,000.00 U/mL with 1:100 dilution). Test results < 0.80 U/mL were considered non-reactive, and those ≥ 0.80 U/mL were considered reactive, with a sensitivity of 98.80% and a specificity of 99.98% [1]. Notably, the assigned U/mL for the assay was equivalent to the binding antibody unit (BAU)/mL defined by the First World Health Organization (WHO) International Standard for Anti-SARS-CoV-2 Immunoglobulin (NIBSC code 20/136) [2].

References

1. Roche Diagnostics GmbH. *Elecsys® Anti-SARS-Cov-2-S, Instructions for Use*; 2021.
2. Jochum, S.; Kirste, I.; Hortsch, S.; Grunert, V.P.; Legault, H.; Eichenlaub, U.; Kashlan, B.; Pajon, R. Clinical Utility of Elecsys Anti-SARS-CoV-2 S Assay in COVID-19 Vaccination: An Exploratory Analysis of the mRNA-1273 Phase 1 Trial. *Front Immunol* **2021**, *12*, 798117, doi:10.3389/fimmu.2021.798117.

Table S1. Characteristics of Patients without Seroconversion after First or Second Dose of Vaccination

Case No.	Age (year)	Sex	BMI (kg/m²)	Etiology of ESKD	HD Vintage (year)	Immunosuppressant or Transplantation	Alb (g/L)	CRP (mg/L)	ACOV2S (U/mL)		
									Week 0	Week 4	Week 8
1	86	Female	27.10	GN	25.16	Nil	41.80	6.33	< 0.40	< 0.40	< 0.40
2	72	Female	24.60	Diabetes	15.90	Nil	39.50	3.33	< 0.40	< 0.40	< 0.40
3	73	Male	22.00	Diabetes	8.57	Nil	36.20	6.01	< 0.40	< 0.40	< 0.40
4	64	Female	19.90	GN	0.12	Transplantation#	40.20	0.59	< 0.40	< 0.40	< 0.40
5	61	Female	22.50	Diabetes	1.48	Nil	37.60	1.47	< 0.40	16.50	6.36
6	73	Male	22.50	Uncertain	18.89	Nil	36.90	1.46	0.75	16.30	8.49
7	52	Male	23.40	Diabetes	5.70	Nil	45.70	2.54	< 0.40	16.70	9.87
8	61	Male	30.30	Diabetes	1.76	Nil	45.60	6.53	0.72	14.30	11.30
9	69	Male	21.90	Uncertain	17.26	Nil	39.80	4.18	0.70	32.90	13.00
10	81	Male	25.70	GN	5.45	Nil	38.10	5.67	< 0.40	38.10	16.90
11	46	Female	18.80	SLE	25.47	Immunosuppressant	39.00	2.08	< 0.40	59.30	22.70
12	72	Male	21.10	Diabetes	2.21	Nil	39.10	12.07	0.78	67.40	25.40
13	45	Male	16.40	Diabetes	2.45	Nil	45.80	2.76	0.40	59.60	26.40
14	86	Female	26.80	GN	0.88	Nil	35.00	3.44	< 0.40	97.00	26.70
15	77	Male	21.00	HTN	14.10	Immunosuppressant	40.80	2.34	< 0.40	44.10	27.20
16	74	Female	21.60	Uncertain	23.76	Nil	40.40	1.39	< 0.40	78.90	31.20
17	71	Male	22.40	HTN	7.86	Nil	42.10	3.39	< 0.40	80.80	46.80
18	68	Female	24.50	GN	23.77	Nil	43.50	7.33	< 0.40	136.00	64.50
19	40	Female	24.90	SLE	4.11	Immunosuppressant	43.80	0.94	0.61	164.00	77.60
20	43	Male	23.30	GN	0.30	Transplantation#	42.20	2.50	< 0.40	282.00	91.70
21	78	Female	23.20	TIN	4.20	Nil	38.00	16.90	0.54	409.00	170.00
22	80	Female	20.70	Diabetes	0.88	Nil	38.80	8.82	< 0.40	468.00	283.00
23	72	Male	19.70	Diabetes	17.84	Nil	35.20	4.58	0.40	397.00	361.00
24	81	Female	19.00	Diabetes	0.13	Nil	35.30	36.13	0.54	961.00	486.00
25	70	Male	28.90	Diabetes	6.11	Nil	37.60	0.61	< 0.40	688.00	589.00
26	52	Female	17.40	SLE	0.90	Nil	39.90	6.40	0.58	839.00	633.00
27	89	Male	24.70	GN	2.21	Nil	35.60	7.21	0.70	1,385.00	906.00
28	62	Male	29.10	PKD	10.08	Nil	41.30	2.93	0.74	2,450.00	1,607.00
29	51	Female	25.80	Uncertain	4.78	Nil	39.80	1.67	0.46	2,125.00	2,234.00

ACOV2S, anti-SARS-CoV-2 spike protein antibody; Alb, albumin; BMI, body mass index; ESKD, end-stage kidney disease; GN, glomerulonephritis; HD, hemodialysis; CRP, C-reactive protein; HTN, hypertension; PKD, polycystic kidney disease; SARS-CoV-2, severe acute respiratory syndrome coronavirus 2; SLE, systemic lupus erythematosus; TIN, tubulointerstitial nephritis. #: kidney transplantation.
